# Using transcriptomics to enable a plethodontid salamander (*Bolitoglossa ramosi*) for limb regeneration research

**DOI:** 10.1186/s12864-018-5076-0

**Published:** 2018-09-25

**Authors:** Claudia M. Arenas Gómez, Ryan M. Woodcock, Jeramiah J. Smith, Randal S. Voss, Jean Paul Delgado

**Affiliations:** 1 Grupo de Genética, Regeneración y Cáncer, Universidad de Antioquia, Sede de Investigación Universitaria, Torre 2, laboratorio 432. Calle 62 No. 52 – 59, Medellín, Colombia; 2 Department of Biology, University of Kentucky, Lexington, KY 40506 USA; 3 Department of Neuroscience, Spinal Cord and Brain Injury Research Center, University of Kentucky, Lexington, KY 40536 USA; 4 Keene State College, Keene, NH USA

**Keywords:** Axolotl, *Bolitoglossa*, Limb, Plethodontid, Regeneration, Transcriptomics, Urodele

## Abstract

**Background:**

Tissue regeneration is widely distributed across the tree of life. Among vertebrates, salamanders possess an exceptional ability to regenerate amputated limbs and other complex structures. Thus far, molecular insights about limb regeneration have come from a relatively limited number of species from two closely related salamander families. To gain a broader perspective on the molecular basis of limb regeneration and enhance the molecular toolkit of an emerging plethodontid salamander (*Bolitoglossa ramosi*), we used RNA-Seq to generate a de novo reference transcriptome and identify differentially expressed genes during limb regeneration.

**Results:**

Using paired-end Illumina sequencing technology and Trinity assembly, a total of 433,809 transcripts were recovered and we obtained functional annotation for 142,926 non-redundant transcripts of the *B. ramosi* de novo reference transcriptome. Among the annotated transcripts, 602 genes were identified as differentially expressed during limb regeneration**.** This list was further processed to identify a core set of genes that exhibit conserved expression changes between *B. ramosi* and the Mexican axolotl (*Ambystoma mexicanum*), and presumably their common ancestor from approximately 180 million years ago.

**Conclusions:**

We identified genes from *B. ramosi* that are differentially expressed during limb regeneration, including multiple conserved protein-coding genes and possible putative species-specific genes. Comparative analyses reveal a subset of genes that show similar patterns of expression with ambystomatid species, which highlights the importance of developing comparative gene expression data for studies of limb regeneration among salamanders.

**Electronic supplementary material:**

The online version of this article (10.1186/s12864-018-5076-0) contains supplementary material, which is available to authorized users.

## Background

Amphibians belonging to the order Caudata (Urodela) have been studied for more than 100 years in developmental biology and more specifically in the area of tissue regeneration [1]. Studies of relatively few species suggest that salamanders, in general, have a broad capacity to regenerate different tissues and organs, including the heart, brain, jaws, tail and the complex structures of complete limbs [2]. However, recent studies have revealed a surprising degree of interspecific variation in regenerative capacity and in the cellular mechanisms by which regeneration is accomplished. For example, satellite cells serve as the progenitors for reforming muscle during regeneration in the Mexican axolotl (*Ambystoma mexicanum*; Family Ambystomatidae) and larvae of the eastern red-spotted newt (*Notophthalmus viridescens*; Family Salamandridae), whereas adult eastern red-spotted newts regenerate using progenitors that are derived from dedifferentiation of functional muscle fibers [3, 4]. These and other examples [5, 6], which are based on relatively few comparative data, suggest that at least some mechanisms of regeneration have diverged during salamander evolution. Clearly, there is need to study additional species to resolve conserved/common mechanisms and taxon-specific differences that have accrued during salamander evolution.

**Fig. 1:**
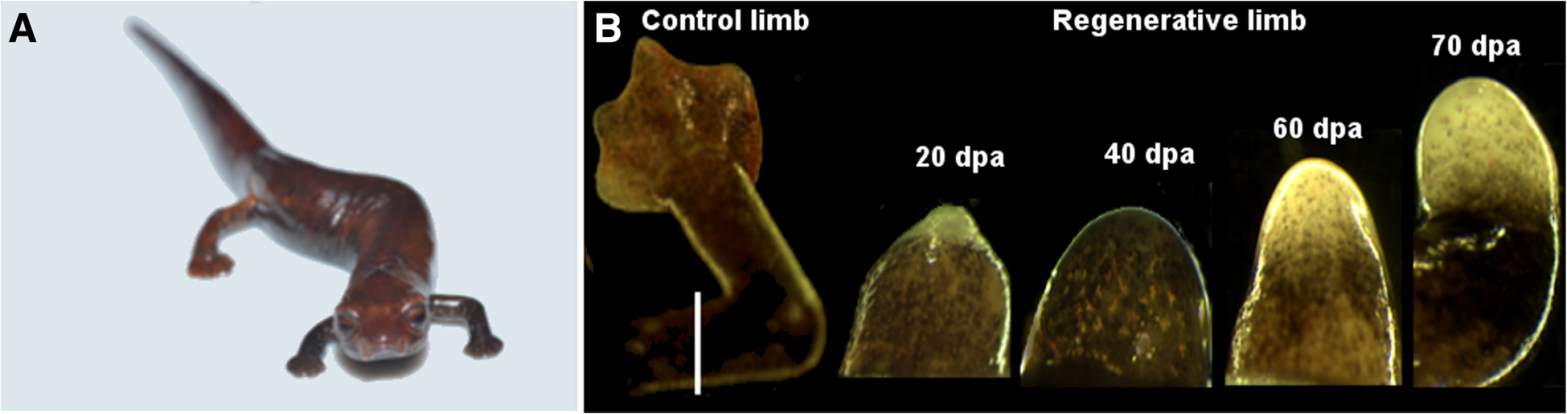
*Bolitoglossa ramosi*. **a** Wild adult salamander of *B. ramosi* (7–10 cm snout to tail) where the forelimbs had completed 28 weeks of regeneration**. b** Tissues used during this analysis. The control limb was the intact limb of the animal; the white line indicates the point of the amputation. The regenerative tissues were the blastema of 20 days post-amputation (dpa), blastema of 40 dpa, blastema 60 dpa, and early palette of 70 dpa

RNA-seq provides an efficient methodology to generate fundamental molecular information for enabling analyses that leverage new model species. To date, transcriptional analyses of limb regeneration have been limited to just a few ambystomatid and salamandrid species (Table 1), with the most transcript data generated for *A. mexicanum* [7, 8] and *N. viridescens* [9]. Transcriptomic data were recently generated for the Chinese giant salamander (*Andrias davidianus*; Family Cryptobranchidae) [10] and Chinese salamander (*Hynobius chinensis*; Family Hynobiidae) [11], but these data were not collected from regenerating tissues. To gain a broader perspective on limb regeneration and salamander evolution in general, we generated transcriptomic data for *Bollitoglossa ramosi* (Fig. 1a)*,* a South American species from the family Plethodontidae. Plethodontid salamanders diverged from all other salamander families approximately 180 million years ago (162.2–199.0 MYA) [12] and exhibit several traits not observed in other salamanders, including enucleated red blood cells, projectile tongues, absence of lungs [13], tail autotomy [14], nasolabial grooves, and postaxial development of the digits [15]. Also, some plethodontids (including *B. ramosi*) undergo direct development, wherein individuals hatch from eggs in the adult form and lack a free-living larval phase [16]. While limb regeneration has been investigated in plethodontid salamanders [17], no study to date has used a transcriptomic approach to globally characterize gene expression.

**Table 1:** Previous studies and tools used to analyze the genes involved during tissue regeneration in salamanders

Reference	Goal	Species	Tissue	Year	Technique
Smith J et al. [57]	EST resource for Ambytomatidae salamanders	*A. mexicanum* *A. tigrinum*	Multiple tissues	2004	Sanger sequencing
Monaghan JR et al. [58]	Gene expression during spinal cord regeneration	*A. mexicanum*	Spinal cord	2007	Microarray
Makarev E et al. [59]	Gene expression during lenses regeneration	*N. viridescens*	Eye	2007	Microarray
Monaghan JR et al. [45]	Transcription during nerve-dependent limb regeneration	*A. mexicanum*	Limb	2009	Microarrays and 454 platforms
Maki N et al. [60]	Gene expression during lenses regeneration	*N.viridescens, Cynops pyrrhogaster*	Eye	2010	Sanger sequencing
Campbell LJ et al. [61]	Gene expression profile of the regeneration epithelium	*A. mexicanum*	Epithelium	2011	Microarray
Holman EC et al. [62]	microRNA expression during limb regeneration	*A. mexicanum*	Limb	2012	Microarray
Monaghan JR et al. [42]	Gene expression during limb regeneration	*A. mexicanum*	Limb	2012	Microarray
Mercer S et al. [38]	Multi-tissue regeneration signature	*N.viridescens*	Multiple tissues	2012	Microarray
Sousounis K et al. [63]	Gene expression during lenses regeneration	*N. viridescens*	Eye	2013	Microarray
Looso M et al. [64]	Tissue regeneration	*N. viridescens*	Multiple tissues	2013	Sanger sequencing, Illumina and 454 platforms
Abdullayev I et al. [65]	Reference transcriptome and proteome during regeneration	*N. viridescens*	Multiple tissues	2013	Illumina
Stewart R et al. [36]	Early gene expression in the blastema	*A. mexicanum*	Limb	2013	Illumina
Wu Ch et al. [46]	Differentially expressed genes during limb regeneration	*A. mexicanum*	Limb	2013	Illumina
Nakamura K et al. [66]	Early Processes of Retinal regeneration	*C. pyrrhogaster* Eyes		2014	Illumina
Voss SR et al. [26]	Global analysis of gene expression Of limb regeneration during 28 days	*A. mexicanum*	Limb	2015	Microarrays
Bryant DM et al. [7]	Axolotl de Novo Transcriptome from multiple tissue to indentify regeneration factors	*A. mexicanum*	Multiple tissues	2017	Illumina
Elewa A et al. [67]	The genome and reference transcriptome of *Pleurodeles waltl*	*Pleurodeles waltl genome*	body parts and regenerative tissues	2017	Illumina
Nowoshilow S et al. [68]	The axolotl genome and Transcriptome from multiple tissue	*A. mexicanum*	Multiple tissues	2018	Different plataforms (PacBio, Illumina)

In the last decade, the use of next generation sequencing platforms have been used to discover novel gene during tissue regeneration

The transcript data that we report for *B. ramosi* follow a recent study that described limb regeneration in this species at anatomical and histological levels [18]. Limb regeneration in *B. ramosi* exhibits morphological similarities to axolotl and newts but also some notable differences, including a more protracted period of blastema growth preceding digit formation, hyperpigmentation of the wound epithelium and collagen deposition in the mesenchymal tissue at 40 days post amputation (dpa). While these differences indicate that regeneration processes are not entirely conserved across divergent salamander families, salamanders, in general, share a blastema-based, developmental strategy to reform amputated limbs. Thus, it seems likely that many core regeneration processes are likely to be shared among salamanders, leading us to perform transcriptome analysis of limb regeneration in *B. ramosi*. We identified genes from *B. ramosi* that are differentially expressed during limb regeneration, including multiple conserved protein-coding genes, noncoding RNAs, and putative species-specific genes. Comparative analyses reveal a subset of genes that show similar patterns of expression in ambystomatid species, identifying these as key probes for identifying conserved mechanisms of limb regeneration.

## Methods

### Animals and surgical procedures

All animals used in this work were caught in the wild in non-private owned land, under the Contract on Access to Genetic Resources number 118–2015, which was delivered by the Ministerio del Medio Ambiente (Ministry of Environment) of Colombia to the Principal Investigator and all experimental procedures were approved by the Institutional Bioethics and Animal Care and Use Committee of the University of Antioquia (Medellín, Colombia). Wild adult salamanders (7–10 cm snout to tail length) of the species *Bolitoglossa ramosi* were collected from their type locality by the nocturnal visual encounter method [19] in the Andes region of Antioquia, Colombia. Specimens were kept in the laboratory under established protocols [20].

A total of 22 adults of *B. ramosi* were administered limb amputations and tissues from the limb, gut, and skin were collected at the time of amputation, and at 20, 40, 60 and 70 days post-amputation (dpa) (Fig. 1b). Briefly, animals were anesthetized by immersion in 1% Tricaine (SIGMA, USA) before bilateral-proximal amputations of the forelimb were performed at mid-humerus level using microscissors and forceps (Fine Precision Tools, USA) [20]. The protruding bone and muscle were trimmed to produce a flat wound surface. Under anesthesia, approximately 2 mm of tissue was collected from the distal tip of amputated limbs. Animals were then euthanized in 2% Tricaine and gut and skin tissues were collected. Overall, we did pools of different animals to get the tissues of controls (D0) (three biological replicates, *n* = 9 animals), gut (one biological replicate, *n* = 1 animal), skin (one biological replicate, *n* = 1 animal), blastema 20 dpa (one biological, replicate *n* = 1 animal), blastema 40 dpa (one biological replicate, *n* = 2 animals), blastema 60 dpa (two biological replicates, *n* = 5 animals) and 70 dpa (one biological replicate, *n* = 3 animals). Samples were collected and stored in TRIzol® reagent until total RNA was extracted using the protocol from the reagent manufacturer (Life Technologies).

### Illumina sequencing

The quality of RNA samples was assessed using an Agilent Technologies 2100 Bioanalyzer. Only samples with an RNA Integrity Number > 8 were used for preparing sequencing libraries. RNA-Seq libraries were prepared from total RNA using TruSeq RNA Sample Prep Kit (Illumina) and the resulting libraries were paired-end (PE) sequenced (2 × 100 bp) using an Illumina Hiseq-2000. The average depth of sequencing for each sample was ~50 million reads (Additional file 1).

### De novo transcriptome assembly

The quality of the raw data was assessed using FastQC [21]. Transcripts were assembled using the Trinity (V 2.0.6) software pipeline [22] and default Trimmomatic parameters to remove sequence adapters and low quality reads (Phred score < 5). In silico reads were further normalized according to the depth of sequence coverage using default settings for Kmer coverage (k = 25). The reference transcriptome was assembled from all RNA samples and all contigs with length ≥ 200 nucleotides were extracted to generate an initial reference transcriptome. Transcriptome assembly quality was assessed based on the calculated E90N50 contig length and BUSCO annotation (Benchmarking Universal Single-Copy Orthologs) [23].

### Gene annotation and GO analysis

Using Reciprocal Best Hits of translation Blast searches (RBH-Blast) [24] with BLASTx and tBLASTn, *B. ramosi* contigs were searched against bacterial, viral, single-celled eukaryote, fungal, salamander ribosomal, and salamander mitochondrial sequence databases compiled from NCBI in order to identify potential contaminants. Sequences with sequence identity ≥50% and bit scores values ≥50 were removed from the reference transcriptome (*N* = 4217). We further predicted long open reading frames (ORF) using TransDecoder (version 3.0.0) software [22]. For gene annotation, *B. ramosi* contigs were reciprocally Blast searched against vertebrate sequence databases to identify high identity alignments that could be used to infer homology relationships. Blast searches were performed using translated nucleotide sequences of *A. mexicanum* [25–27] and *N. viridescens* [9], and protein-coding sequences from seven vertebrate taxa (*Anolis carolinensis:* GCA_000090745.1, *Danio rerio:* GCA_000002035.3, *Gallus gallus:* PRJNA10808, *Homo sapiens:* PRJNA168, *Mus musculus:* GCA_000001635.7, *Xenopus tropicalis:* PRJNA205740, *Latimeria chalumnae*: GCA_000225785.1*;* available through Ensembl or Refseq NCBI). Gene names were assigned to *B. ramosi* contigs if an alignment showed ≥50% identity and returned a bit scores value ≥50.

### Transcript abundance (RSEM) and expression level analyses (EBSeq)

Sequence reads generated from limb tissue samples were aligned to the reference transcriptome using Bowtie2 [28] and RSEM (RNA-Seq by Expectation Maximization) was used to obtain estimates of transcript abundance for all transcripts [29]. Expression levels were calculated as transcripts per million (TPM). For cases where two or more significant transcripts mapped to the same gene identifier, only the transcript with the highest expression estimate was evaluated statistically using EBseq. Transcripts were considered differentially expressed between control and post-amputation samples when TPM was ≥0.95 (4 to 800,000 expected counts) for at least a single time point and fold change (log2FC) was ≤ − 2 and ≥ 2 with an FDR *p* < 0.05 (Fold discovery rate). Differentially expressed genes were further analyzed using PANTHER gene expression tools (Version 11.1) to identify corresponding Gene Ontology (GO) terms [30].

### RT-qPCR

Three replicate tissue samples from collected from unamputated and regenerating (40 and 60 days post amputation) forelimbs and total RNA was prepared using TRIzol® reagent (Life Technologies). The total RNA was reverse-transcribed to single-stranded cDNA with reverse transcriptase (Thermo) in the presence of random hexamer primers, oligoDT primers, and dNTPs for 60 min at 42 °C. Expression levels of specific mRNAs were determined by qPCR using gene-specific primer pairs (Additional file 2) (two technical replicates). Each reaction was performed at a total volume of 10 μL containing 50 ng first-strand cDNA, 5 μL Syber greenMix (Biorad), and 0.1 μM of each primer pair, and cycled on a Biorad Real-Time PCR system. Real-time data were analyzed using Biorad software version 2.1. Relative mRNA expression was calculated using the 2 ^–ΔΔCT^ method with GAPDH as a cross-sample reference. Significance was determined using a two-tailed Unpaired T-test and Welch’s correction (*P* < 0.05). Pearson correlation (*P* < 0.05) was performed to analyze the correlation between the log2 Fold change of 40dpa and 60dpa (relative to the control unamputated sample) obtained in the in silico analyses by RNA-Seq and with the validation by RT-qPCR.

## Results

### Transcriptome overview

A de novo reference transcriptome of *B. ramosi* was generated from RNAs that were isolated from normal limb, regenerating limb, skin, and gut. The total number of high quality assembled PE reads recovered was 654,673,506. Using Trinity software, we obtained 433,809 contigs with an average GC content of 43.5%, an average length of 569 bp, and a maximum assembled contig length of 20,709 bp (Table 2). On the basis of read coverage, the E90N50 statistic was ~3 Kb (Additional file 3) and the reference transcriptome contained 85.1% of the conserved core eukaryotic genes using BUSCO annotation (Additional file 4). Blast searches of *B. ramosi* contigs identified 36 mitochondrial, zero rDNA, and 4181 putative microorganism hits. Notably, multiple hits (*n* = 66) matched sequences from the pathogenic chytrid fungus *Batrachochytrium dendrobatides.* This fungus is a primary cause of worldwide declines in amphibian populations [31] and was first described in Colombia in 2013 [32].

**Table 2:** Trinity assembly summary statistics of de novo reference transcriptome for limb regeneration in a non-model terrestrial salamander, *Bolitoglossa ramosi* (Caudata: Plethodontidae)

Parameter	Number
Total aligned reads	1,641,919,128
Total number of high quality assembled paired-end reads	654,673,506
Total trinity transcripts	577,037
Total trinity ‘genes’	433,809
Average ‘genes’ length (pb)	569
%GC	43,59
Longest contig (bp)	20,709
Shortest contig	224
Number of contigs > 200 bp	390,662
Number of contigs > 1 Kb	40,133
Number of contigs > 5 Kb	2797
Number of contigs > 10 Kb	217
Number of predict ORFs (transdecoder)	83,764

**Fig. 2:**
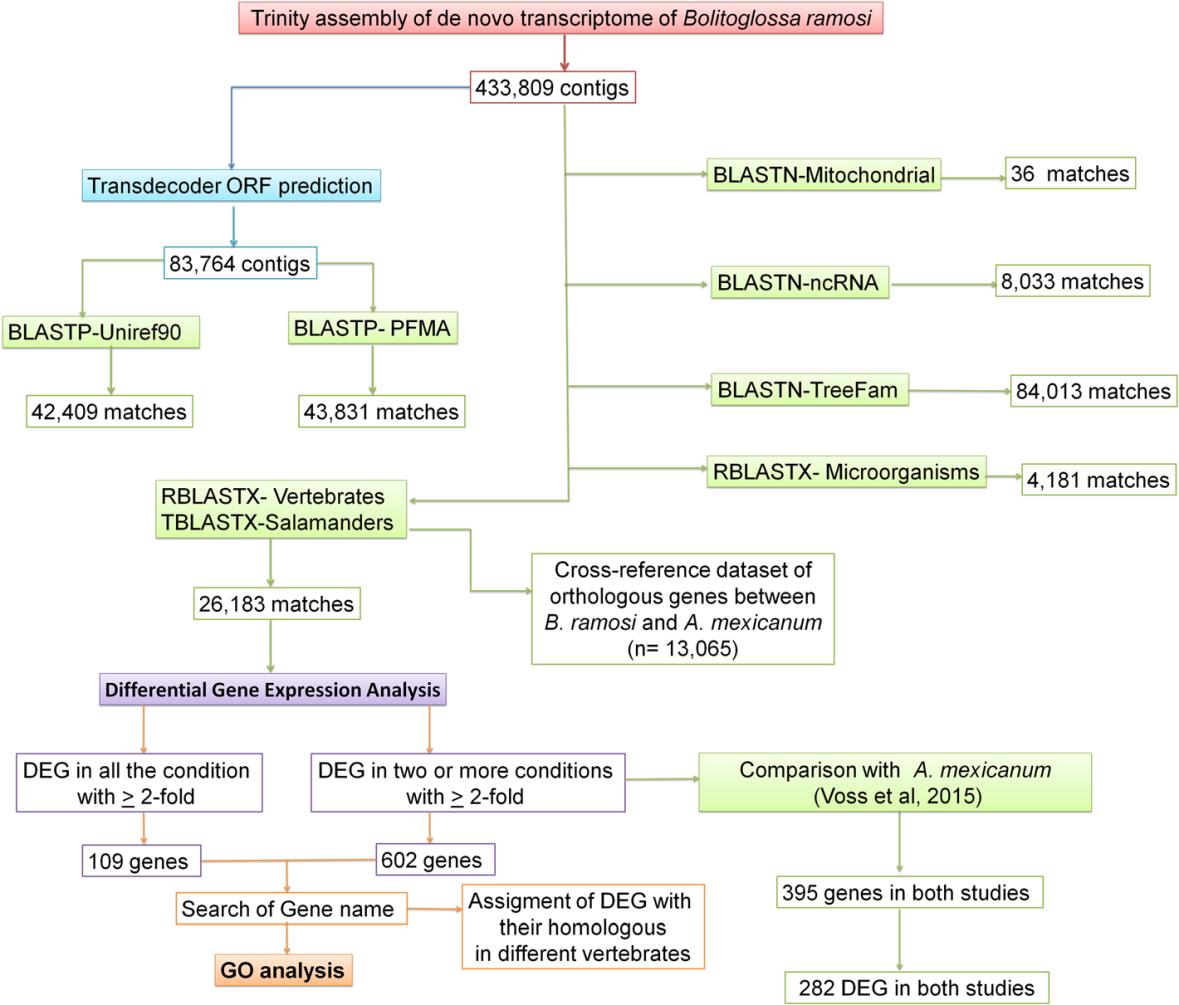
Flowchart of strategies used to annotate the reference trancriptome of *Bolitoglossa ramosi*. Different strategies were used to identify homologous genes from different vertebrate and salamander databases. The objective of these analyses was to obtain a gene list of differential expressed genes (DEG) during limb regeneration that could be compared to DEG reported for *Ambystoma mexicanum* [26]

The contigs of *B. ramosi* were annotated by performing BLAST searches against *A. mexicanum* transcriptome databases (Ambystoma.org and Axolotl-omics.org) and *N. viridescens*, and by generating a cross-referenced dataset of orthologous genes between *A. mexicanum* and *B. ramosi* (*n* = 13,065) (Additional file 5). Large divergences were expected between salamanders species (180 MYA), the frequencies of the % identity between *B. ramosi* vs *N. viridescens* and *B. ramosi* vs *A. mexicanum* during the RHB-Blast showing than the major of the matches were > 80% (Additional file 6). We also searched Ensembl and NCBI protein sequences from seven vertebrate taxa, which identified gene annotations for 26,183 *B. ramosi* contigs (Additional file 7). Finally, we obtained homology information for contigs that did not return a sequence match by searching Treefam [33], UniRef90 [34], PFAM [35] (Additional file 8) and ncRNA (miRBase and RFam) [34, 35] (Additional file 9) databases. These searchers (Additional file 10) returned non-redundant annotations for 140,974 of *B. ramosi* contigs. Across all search strategies (Fig. 2), we obtained gene and RNA annotations for 142,926 non-redundant transcripts of the *B. ramosi* reference transcriptome.

### Differential gene expression analysis

**Fig. 3:**
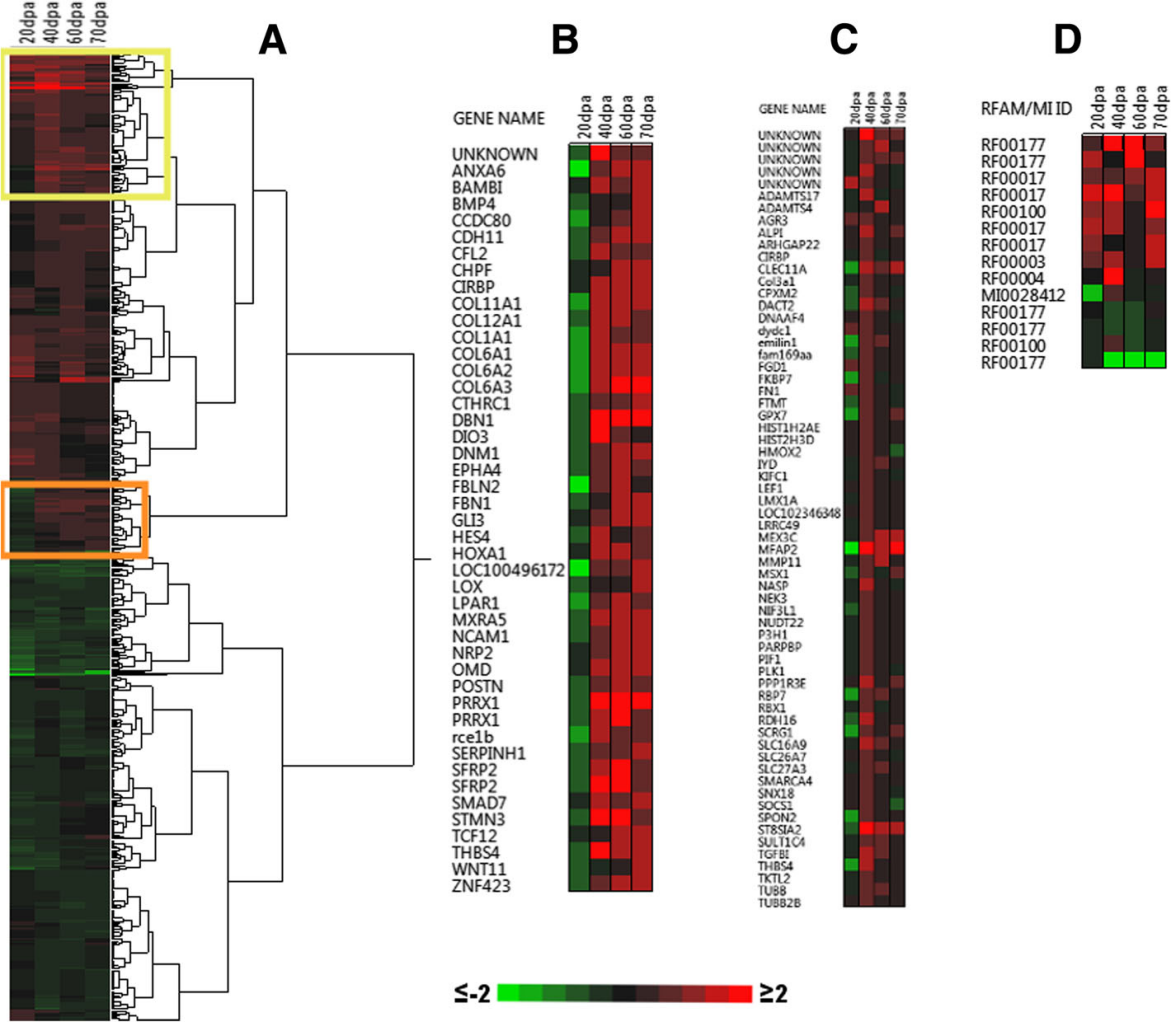
Heatmap of differentially expressed genes (DEGs) during limb regeneration in *Bolitoglossa ramosi* (Caudata: Plethodontidae). **a** DEGs with a ≥ 2-fold expression change and good transcriptional support (TPM ≥ 0.95) were considered (*n* = 602). Each column of the heatmap indicated the 2-fold changes of each sample respect the control limb, dpa: days post-amputation. Two clusters were identified that largely consisted of up-regulated (red clusters, *n* = 310) and down-regulated genes (green cluster, *n* = 292). **b** A group of genes exhibiting down-regulation at 20 dpa but up-regulation from 40 to 70 dpa (orange square in **a**). **c** A group of genes markedly up-regulated at 40 dpa (yellow Square in **a**). **d** ncRNAs identified as DEG, with the heatmap showing RFAM or miRBase ID

Due to limitations in obtaining source materials from wild *B. ramosi*, our study included three replicate samples for the D0 and two replicates for 60 dpa time points, but only single replicate samples for 20, 40, and 70 dpa time points. Although a previous transcriptional study of salamander limb regeneration reported genes as significant from contrasts of single replicates [36], we conservatively only report genes that exhibited statistically significant changes in expression between the control and two or more post-amputation time points, and where the magnitude of the expression difference was > 2-fold (Fig. 3). We reasoned that this approach reduced the number of false positives and differentially expressed genes associated with individual-specific differences (e.g. age, disease history, physiological state) that are unrelated to limb regeneration. Overall, 602 non-redundant differentially expressed genes were identified, of which 556 were assigned an official gene symbol or locus identifier from a model organism database (Additional file 11). Hierarchical clustering identified two primary clusters, one where genes were expressed more highly in regenerating limbs than controls across a majority of time points (*n* = 310) and another where genes were expressed more highly in controls (*n* = 292) (Fig. 3a). The cluster of highly expressed genes in regenerating limbs included extracellular matrix (different collagen isoforms, *emilin1*, *adamts4*, *spon2*, *adamts17*, *mfap2*, *clec11a*, *p3h1*) and developmental process (*lef1*, *msx1*, *sox12*, *sall4*) genes. The cluster of lowly expressed genes in regenerating samples encode transcription factors (*nfkbiz, fhl1, znf385a, tsc22d1, nfil3, sfmbt2, fhl2, pdlim7, nr1d1, foxk2, per1, tfcp2, elf5, znf618, en1, ubp1, foxq1),* and extracellular matrix components (*ogn, mgp, mfap5, slitrk6, crim1, ap1m1, dpt*).

Genes were primarily down-regulated during the earliest post-amputation time point (20 dpa) (Fig. 3b). These genes are associated with collagen biosynthesis (*Col1a1*, *Col6a2*, *Col11a1*, *Col12a1*, *Col6a1*, *Serpinh1*, *Col4a5*, *Col6a3*), axon guidance (*Epha4*, *Nrp2*, *St8sia2*, *Kif4a*, *Ncam1*, *Shb*, *Dnm1*, *Robo1*) and developmental regulation (*Hoxa1*, *Ezh2*). Genes that were up-regulated at 20 dpa showed decreased expression at subsequent sample times, and this list included genes that function in actin crosslinking (GO:0051764) (*Rac2*, *Lcp1*), regulation of the p38MAPK cascade (GO:1900744) (*Per1*), and hemidesmosome assembly (GO:0031581) (*Krt14*).

We note that 109 of the 602 genes were differentially expressed at all post-amputation time points in comparison to the non-amputated condition (Additional file 12), and many of these encode proteins for development processes (45.8%), including *prrx1, sall4, snai2, sox12, pak1, lef1, lefty2,* and *smarca4*.

Some of the genes exhibited complex patterns of gene expression. For example, some genes were expressed differently in regenerating limbs relative to controls at 20 dpa and 40 dpa; these included collagen (*col11a1*, *col1a1*, *col6a1*) and homeobox (*prrx1*, *hoxa1)* genes that were expressed lower than controls at 20 dpa but higher at 40 dpa (Fig. 3b). Also, some genes were more highly expressed in regenerating samples than controls specifically at 40 dpa (Table 3, Fig. 3c), including transcription factors (*msx1*, *lmx1a*), cell adhesion molecules (*tgfbi*), extracellular matrix components *(emilin1, adamts4, spon2, adamts17, mfap2, clec11a, p3h1),* cytoskeletal molecules (*tubb, kifc1, tubb2b, lmx1a)* and developmental process genes (*fgd1, adamts4, spon2, rbp7, lef1, clec11a, alpi, msx1, lmx1a, dact2).*

**Table 3:** Biological processes identified from DEGs identified at 40 dpa during limb regeneration in *Bolitoglossa ramosi* (*p*-value < 0.05)

GO biological process	Matches	*P*- value
developmental process (GO:0032502)	182	7.09E-13
cellular developmental process (GO:0048869)	126	1.33E-08
cell differentiation (GO:0030154)	124	1.67E-08
collagen catabolic process (GO:0030574)	15	4.21E-08
collagen metabolic process (GO:0032963)	16	5.58E-08
extracellular matrix organization (GO:0030198)	27	9.53E-07
extracellular structure organization (GO:0043062)	27	1.02E-06
regulation of developmental process (GO:0050793)	87	8.61E-06
ossification (GO:0001503)	22	5.22E-05
response to stimulus (GO:0050896)	208	9.11E-05
skeletal system development (GO:0001501)	30	1.01E-04
blood vessel development (GO:0001568)	29	3.19E-04
nervous system development (GO:0007399)	78	1.00E-03
osteoblast differentiation (GO:0001649)	13	9.15E-03
embryonic morphogenesis (GO:0048598)	29	1.45E-02
embryo development (GO:0009790)	40	1.55E-02
epithelium development (GO:0060429)	44	1.60E-02
connective tissue development (GO:0061448)	16	1.86E-02
regulation of cell differentiation (GO:0045595)	59	2.28E-02
extracellular matrix disassembly (GO:0022617)	10	3.41E-02
skeletal system morphogenesis (GO:0048705)	16	4.10E-02

The annotated gene list (*N* = 556) was subjected to a statistical over-representation test using Panther Gene List Analysis tools and default settings [30] (Additional file 13). We identified enriched biological process gene ontologies associated with extracellular matrix components, cell differentiation, migration, proliferation, morphogenesis, and development of multiple tissues (epithelium, neurons, vasculature, cartilage, and bone). Many of these genes encode proteins that function in cell signaling pathways associated with tissue development, including Wnt (*wnt11, wnt5a, tcf7l1, wnt4, fzd1, lef1, en1, tp53, smarca4*), TGFb/BMP (*fosl1, smad7, bambi, bmp4, junb*), Hedgehog (*gli3*), Hox (hoxa1), and Notch (*pofut1, hes1, hes4*) (Fig. 3b, c).

**Fig. 4:**
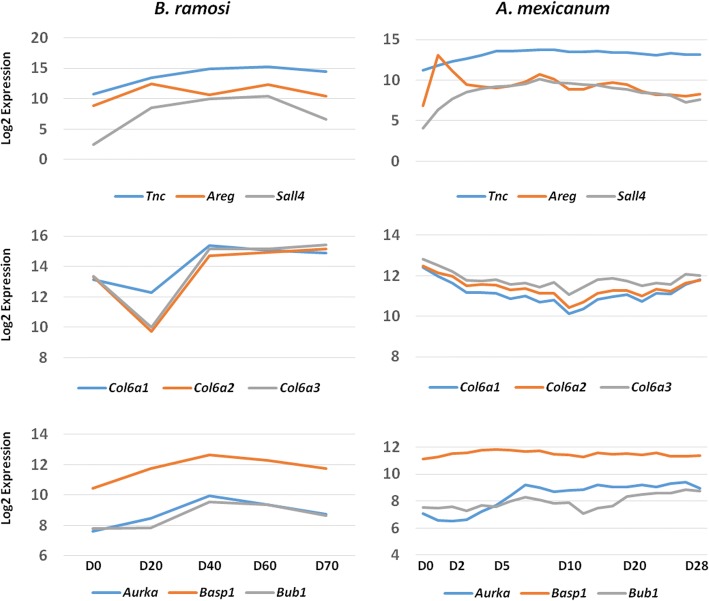
Conservation of gene expression between *Bolitglossa ramosi* (Caudata: Plethodontidae) and *Ambystoma mexicanum* (Caudata: Ambystomatidae). A total of 273 significant genes were expressed similarly between the species. Representative expression profiles are shown for nine of the most highly correlated genes: *Tnc*, *Areg*, *Sall4*, *Col6a1*, *Col6a2*, *Col6a3*, *Aurka*, *Basp1*, *Bub1*. The log_2_ expression values were derived from the expected counts as calculated by RSEM. Values on the X axis reflect time points (post-amputation) evaluated in each study

The significant *B. ramosi* DEGs were further compared against 3053 significant genes identified by Voss et al. [26], the most comprehensive and statistically powered study of *A. mexicanum* limb regeneration to date. In that study, an Affymetrix microarray was used and thus estimates were obtained for a finite number of genes. We determined that the [26] study generated *A. mexicanum* transcript abundance estimates for 395 of the 602 *B. ramosi* DEG genes. The majority (71%; *N* = 282) of these *A. mexicanum* genes were significantly differentially expressed in the [26] study and showed a similar temporal pattern of expression relative to orthologous *B. ramosi* DEGs (Fig. 4). The DEGs shared between *B. ramosi* and *A. mexicanum* were enriched for GO terms associated with protein binding (GO:0005515) (*sall4*, *sox12*, *fhl1*, *pdlim7*), extracellular matrix organization (GO:0030198) (*vcan*, *col6a2*, *col11a1*, *col6a1*, *tnc*, *col6a3*, *mmp1*) and developmental growth (GO:0048589) (*wnt5a*, *aurka*, *sema3f*) (Additional file 14). Also, we found that 104 genes had an ortholog in the axolotl-omics.org database but not in the Voss et al. study (2015) and some genes (*n* = 103) had no ortholog in any of the axolotl databases (Additional file 15).

Finally, we recovered a group of transcripts (*n* = 85) that had good transcriptional support (TPM ≥ 0.95) but did not match sequences from any of the reference databases that were searched. From this group of anonymous transcripts, we identified 11 potentially novel, taxon-specific genes with complete ORFs that were differentially expressed during regeneration. We also identified 14 hits with homology to ncRNAs (Additional file 16). These genes were found to have good transcriptional evidence (TPM ≥ 0.95) and were differentially expressed in some of the conditions evaluated. These candidates included matches to ncRNAs such as eca-mir-9182 (MI0028412), signal recognition particle RNA (RF00017), and 7SK (RF00100) (Fig. 3d).

### Validation by RT-qPCR

**Fig. 5:**
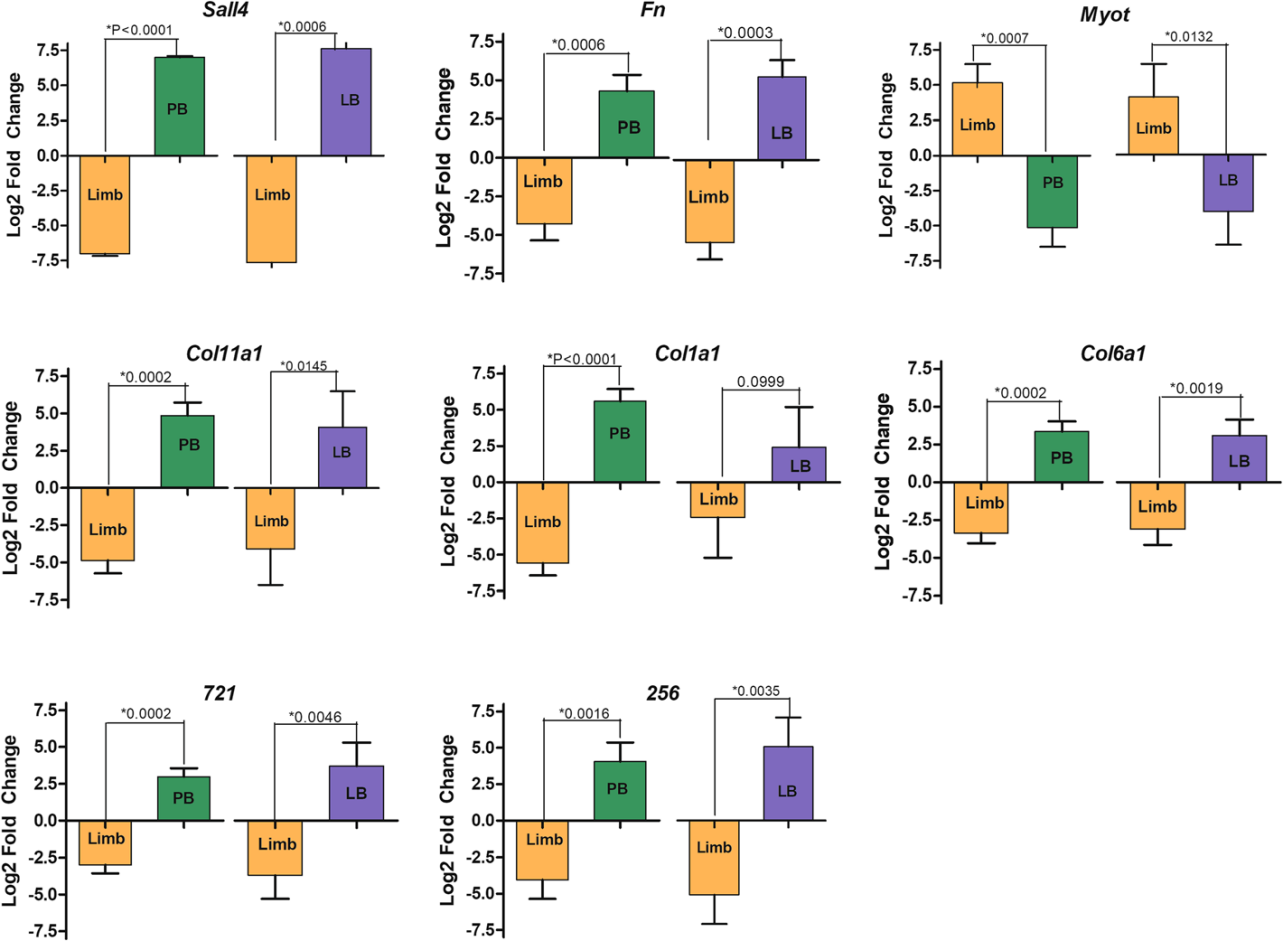
RT-qPCR validation of differentially expressed genes in *Bolitoglossa ramosi* (Plethodontidae). Eight genes were evaluated by RT-qPCR to validate the DEG analysis in silico at 40 days post amputation (dpa) and 60 dpa against the control limb (D0). Bars represent mean ± SD of three independent measurements. The sample means (Control vs 40dpa and Control vs 60dpa) differed significantly (*) under a t-test and 0.05 p-value threshold

We made use of RT-qPCR to validate the expression estimates obtained by RNA-Seq for selected transcripts (*sall4*, *fn1*, *myot, coll11a1, col1a1, col6a1*) (Fig. 5, Additional file 17) that are known to be modulated during limb regeneration [25, 36, 37]. Also, we validated the expression of two *B. ramosi* taxon-specific transcripts. For these validations, biological replicate RNA samples were prepared for control limbs, and for regenerating forelimb tissues collected 40 and 60 dpa. We observed that *sall4*, *fn1*, *col11a1*, *col1a1*, *col6a1* (higher in regenerating limbs) and *myot* (lower in regenerating limbs) showed the same pattern of expression in qPCR as was found using RNA-Seq. Also, the transcripts that we defined as taxon-specific showed the same patterns using both methodologies (Additional file 18).

## Discussion

Here we present the first de novo reference transcriptome of limb regeneration for *B. ramosi,* a salamander belonging to the largest family of salamanders. Plethodontids of the genus *Bolitoglossa* exhibit some notable biological differences in comparison to model salamanders (axolotls and newts) from other taxonomic families, including their restrictive terrestrial habits and direct development without larval stages [14, 16]. We recently reported anatomical and morphological changes during limb regeneration in *B. ramosi* [18]. Many of the changes that we observed, including processes associated with wound healing, blastemal formation, and re-patterning, also occur during limb regeneration in other salamanders. These features are likely conserved among all salamanders and indeed we identified DEGs in *B. ramosi* that are known to be expressed during limb regeneration in *A. mexicanum* and *N. virdescens* [36–38]. These include genes and proteins that associate with the wound epidermis, extracellular matrix, basement membrane, blastema and differentiating chondrogenic precursor cells. Additionally, we identified 109 genes that were differentially expressed throughout regeneration in *B. ramosi*, of which 77 were previously identified as differentially expressed during limb regeneration in *A. mexicanum* [25, 39, 40–42]*.* However, we found genes that were DEG in *B. ramosi* but have not been reported yet as DEG in *A. mexicanum* and *N. virdescens*. This is the case for TGF-beta signaling pathway genes (*Lefty2* and *Bmp4*). The gene *Lefty2* was highly expressed during all time points of regeneration, including at 40 dpa, when the blastema shows increased pigmentation. This gene is implicated in left-right axis determination during development, however, it will be important to further analyze the *Lefty2* function during limb regeneration. Also, *Bmp4* function is important during limb development in mice [43] and direct development of frogs, where it provides an important biomarker of skeletogenic cell differentiation [43, 44]. This gene was up-regulated in *B. ramosi* at 70 dpa, which correlates to the palette stage of limb regeneration when limb skeletal patterning occurs. Thus it is possible that this gene has an important role during regeneration of salamanders with direct development. Collectively, these results suggest an important role for that the TGF-beta signaling pathway during limb regeneration in *B. ramosi.*

Through additional gene expression profile comparisons between *B. ramosi* and *A. mexicanum,* we observed conservation of temporal gene expression patterns (Fig. 4). Many genes that were up-regulated or down-regulated throughout limb regeneration in *B. ramosi* showed the same expression pattern in *A. mexicanum*, and this correlation became even greater when ignoring changes in gene expression at early post-amputation time points in *A. mexicanum* that were not sampled for *B. ramosi*. We note though that it is difficult to reconcile gene expression similarity for some genes, even when considering differences in temporal sampling. For example, *col6a1*, *col6a2*, and *col6a3* show different patterns of expression between *A. mexicanum* and *B. ramosi*, however, their correlated expression within species strongly suggests conservation of mechanisms that regulate transcription during limb regeneration.

Although our results generally support the idea that many aspects of the limb regeneration process are highly conserved among salamander limb regeneration programs, regeneration differences [4, 6, 45, 46] are likely to evolve among lineages that present different modes of development [47] and life history [48, 49]. For example, ECM (Extracellular matrix) proteins are important modulators of cell proliferation, migration, and differentiation, and they also provide structural support to regenerating limbs that is important for blastemal cell survival [50, 51]. The ECM is predicted to be more rigid in regenerating limbs of terrestrial salamanders that support body weight during locomotion. Less rigidity is predicted for aquatic salamanders that use buoyancy to reduce load-bearing on limbs during regeneration, and swimming for locomotion. Consistent with these predictions, we observed high expression of collagen genes during *B. ramosi* limb regeneration and this correlated with a high abundance of collagen protein in the blastema [18]. Generation of a more rigid ECM, perhaps to minimize compressive forces on blastemal cells, may be characteristic of terrestrial salamanders that continue to walk during their protracted, limb regeneration programs. Also, we identified DEGs at 20 dpa that further support this idea; the proteins encoded by *Rac2*, *Lcp1*, and *Krt14* might confer mechanical support to the epithelium and protect the early blastema.

We also note the possibility that some features unique to *B. ramosi* may be associated with taxon-specific genes, a feature that is also known for other salamanders [52–54]. Our analyses identified 12 presumptive taxon-specific genes, two of which were validated using RT-qPCR for control limbs, and 40dpa and 60dpa time points (Fig. 5). All of these transcripts had ORFs > 1000 bp, and 3D prediction with I-TASSER [55] identified peptide sequences consistent with protein folding (primarily alpha helices). It is possible that these represent novel, taxon-specific proteins that could play an important role in *B. ramosi* limb regeneration. Future work will endeavor to determine the function of these genes in vivo.

We would further like to acknowledge that some limitations were encountered in the use of a wild-caught species as a research model. Mandatory environmental licensing was required for the collection of wild specimens, and while these regulations are in the best interests of preserving biodiversity, specimen sampling may be reduced below thresholds needed to perform a high-powered statistical analysis. Indeed, such conditions may present an unfortunate hindrance of biodiversity research in developing countries [56]. The use of wild-caught animals is additionally complicated where standardization of optimal captive conditions may be unknown or difficult to replicate, and where suboptimal conditions place the animals under sustained stress leading to disease and decreased survivorship. Further study of the reproductive biology of *B. ramosi* is needed to establish a captive breeding program and in turn to grant experimental access to more animals of different stages to further test hypotheses generated during this study.

## Conclusions

In this study, we report the first de novo reference transcriptome during limb regeneration in a plethodontid salamander, which allowed the identification of differentially expressed genes. Our study shows conservation of transcriptional regulation across plethodontids and ambystomatids which diverged some 180 Ma ago [12]. The genes that we report should be especially good biomarkers for future comparative studies of limb regeneration among salamander taxa.

## Additional files

Additional file 1: Transcript sequence depth for samples used in this study. (DOCX 11 kb)

Additional file 2: RT-qPCR primers used for validation of gene expression during regeneration in *Bolitoglossa ramosi*. (DOCX 12 kb)

Additional file 3: E90N50 statistic for de novo reference transcriptome of *Bolitoglossa ramosi* . The reference transcriptome has an E90N50 of ~3kb (red arrow). (TIF 221 kb)

Additional file 4: BUSCO Analysis of Transcriptome Completeness. (DOCX 11 kb)

Additional file 5: Output of BLASTn alignments between the *Bolitoglossa ramosi* reference transcriptome, *Ambystoma mexicanum* and *Notophthalmus viridescens* databases. (XLSX 16976 kb)

Additional file 6: Histogram that shows the frequencies of the % identity between *B. ramosi* vs *N. viridescens* and *B. ramosi* vs *A. mexicanum* during the RHB-Blast. (TIF 3750 kb)

Additional file 7: Output of the RBH-BLAST alignments of the *Bolitoglossa ramosi* reference transcriptome using seven vertebrate databases. (XLSX 36198 kb)

Additional file 8: Output from the BLASTp alignments of the *Bolitoglossa ramosi* predicted ORFs against TreeFam, PFAM and UniRef90 databases. (XLSX 73915 kb)

Additional file 9: Output from the BLASTn alignments of the *Bolitoglossa ramosi* reference transcriptome against the ncRNA database. (XLSX 950 kb)

Additional file 10: Homology assignments recovered in de novo reference transcriptome assembly of *Bolitoglossa ramosi* . The *B. ramosi* transcriptome was surveyed by Reciprocal Best Hits of translated BLAST searches (RBH-BLAST) to protein or translated databases from different vertebrates. Additional gene family homologs were assigned to *B. ramosi* using protein BLAST against the UniRef90, TreeFam and PFAM domain databases, as well as BLASTN against ncRNA databases. (TIF 48 kb)

Additional file 11: DEGs identified from *Bolitoglossa ramosi* with ≥2-fold (Log_2_) difference relative to the unamputated control at two or more post-amputation time points, and with TPM support ≥0.95. (XLSX 227 kb)

Additional file 12: List of 109 genes that were differently expressed at all post-amputation time points in comparison to the non-amputated condition. (XLSX 31 kb)

Additional file 13: Statistical over-representation test of biological processes using Panther Gene List Analysis tools of the annotated gene list. (XLSX 13 kb)

Additional file 14: Enriched GO associated terms for DEGs shared between *Bolitoglossa ramosi* and *A. mexicanum*. (XLSX 18 kb)

Additional file 15: List of DEG of *B. ramosi* not reported yet as differential expressed genes during limb regeneration in *A. mexicanum*. (XLSX 8 kb)

Additional file 16: Transcripts annotated as possible ncRNA which were differentially expressed during limb regeneration in *Bolitoglossa ramosi*. (XLSX 8 kb)

Additional file 17: RT-qPCR output and comparison of RT-qPCR and RNA-Seq estimates of gene expression. (XLSX 26 kb)

Additional file 18: Correlation between RNA-Seq and quantitative real time PCR (RT-qPCR). The expression patterns are similar between the RNA-Seq and RT-qPCR data, and statistically significant Pearson correlation is shown for the expression levels of eight genes measured with both methodologies during 40 dpa and 60 dpa of limb regeneration. The X and Y axis show log_2_ fold change for RT-qPCR and RNA-Seq values, respectively. (TIF 133 kb)

## Abbreviations

BUSCO: Benchmarking universal single-copy orthologs
D0: Day zero, control limb
DEGs: Differential expressed genes
dpa: Days post amputation
ECM: Extracellular matrix
FC: Fold change
FDR: Fold discovery rate
GO: Gene ontology
MYA: Million years ago
ORF: Open reading frames
PE: Paired end
RBH-Blast: Reciprocal best hits of translation blast searches
RSEM: RNA-seq by expectation maximization
TPM: Transcripts per million
