# Supplementary material for: Using transcriptomics to enable a plethodontid salamander (Bolitoglossa ramosi) for limb regeneration research
Source: BMC Genomics. 2018 Sep 25;19:704. doi: 10.1186/s12864-018-5076-0 (PMC6157048; doi:10.1186/s12864-018-5076-0)
Supplement: Supplementary file 1 — Transcript sequence depth for samples used in this study. (DOCX 11 kb) [file 12864_2018_5076_MOESM1_ESM.docx]

| Sample | # of sequence reads |
| --- | --- |
| limb | 55,128,678 |
| limb | 60,829,288 |
| limb | 59,029,316 |
| Regenerative tissue | 50,594,572 |
| Regenerative tissue | 60,253,528 |
| Regenerative tissue | 50,665,182 |
| Regenerative tissue | 46,787,228 |
| Regenerative tissue | 47,509,576 |
| Gut | 62,583,024 |
| Skin | 38,979,054 |
