# Supplementary material for: Using transcriptomics to enable a plethodontid salamander (Bolitoglossa ramosi) for limb regeneration research
Source: BMC Genomics. 2018 Sep 25;19:704. doi: 10.1186/s12864-018-5076-0 (PMC6157048; doi:10.1186/s12864-018-5076-0)
Supplement: Supplementary file 2 — RT-qPCR primers used for validation of gene expression during regeneration in Bolitoglossa ramosi. (DOCX 12 kb) [file 12864_2018_5076_MOESM2_ESM.docx]

| Gene | Forward Primer (5'-3') | Reverse Primer (5'-3') |
| --- | --- | --- |
| *Myot* | CGC TTT CTT CCT CCT CTT CA | AGG CTC TCG GAT TCT GTG TG |
| *Sall4* | AGG GTG ACT GTG CTT TGG AC | GCT GGT GGT AGG CGA AGA TA |
| *Gapdh* | AAC ATC ATT CCA GCC TCC AC | GCA GCC TTC ACA ACC TTC TT |
| *Fn1* | GCA GCC AGT TGA AGA CAC CT | CCC ATT TCT CAC CAA TCC TG |
| *Col11a1* | TAG TAA AGG GAG AGT TAG GCG AAT A | CTT GTT TCT GTG ATG TCC GTC |
| *Col1a1* | TAG CGG TCC AAA AGG TGA AC | CAA TAC CAC GAG AAC CAA CTG |
| *Col6a1* | CGA CAC CAT CAA AGC AAA CG | CAC CCT CAT AAC CTG GAT CAC |
| *T721* | GGT GCC TGT AAG CGG AGA | GAC CCT CGT GTT CCT TTT CC |
| *T256* | CGG CAA ACA AAC ACG ACT | GAG GGA AAG GAA AGG ACA GAG |
