# Supplementary figures and images for: Using transcriptomics to enable a plethodontid salamander (Bolitoglossa ramosi) for limb regeneration research
Source: BMC Genomics. 2018 Sep 25;19:704. doi: 10.1186/s12864-018-5076-0 (PMC6157048; doi:10.1186/s12864-018-5076-0)

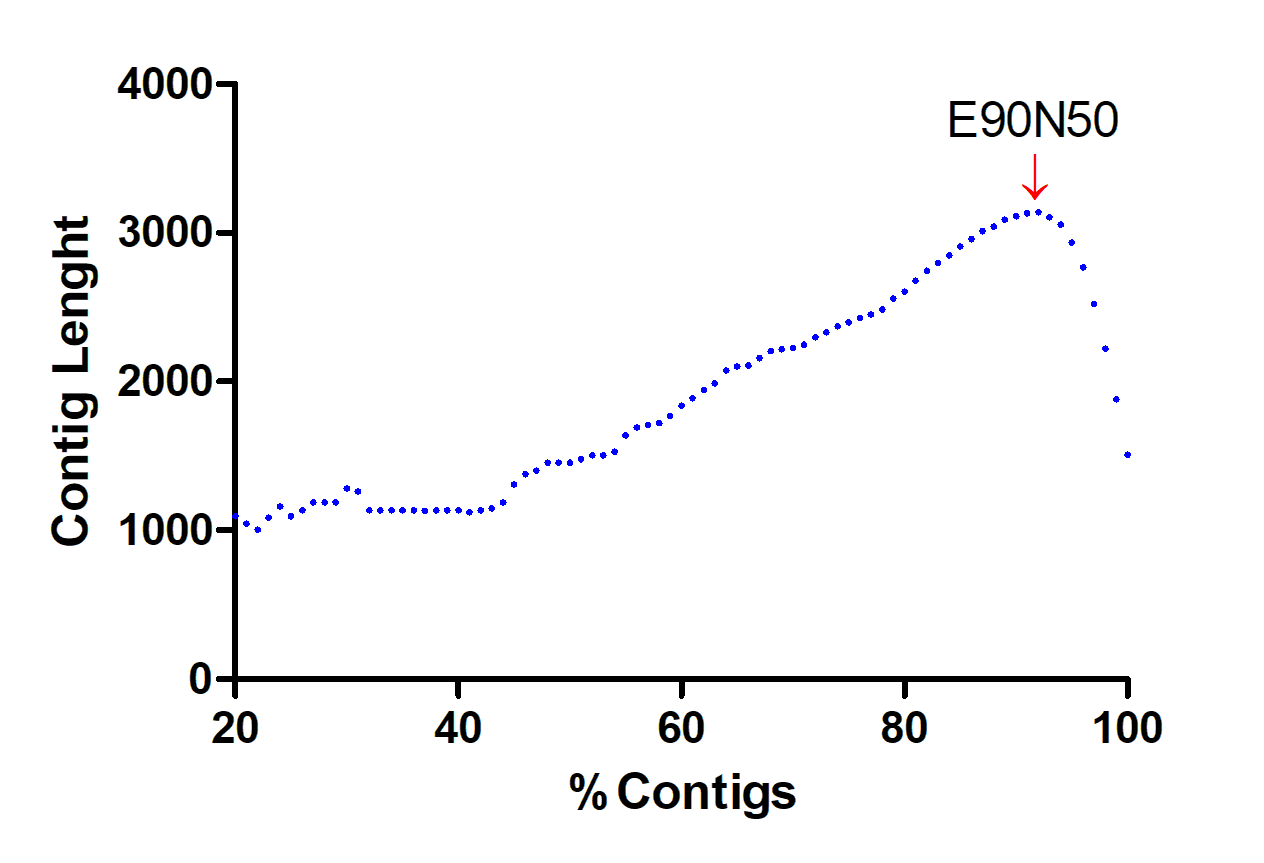

Supplement: Supplementary file 3 — E90N50 statistic for de novo reference transcriptome of Bolitoglossa ramosi . The reference transcriptome has an E90N50 of ~3kb (red arrow). (TIF 221 kb) [file 12864_2018_5076_MOESM3_ESM.tif]

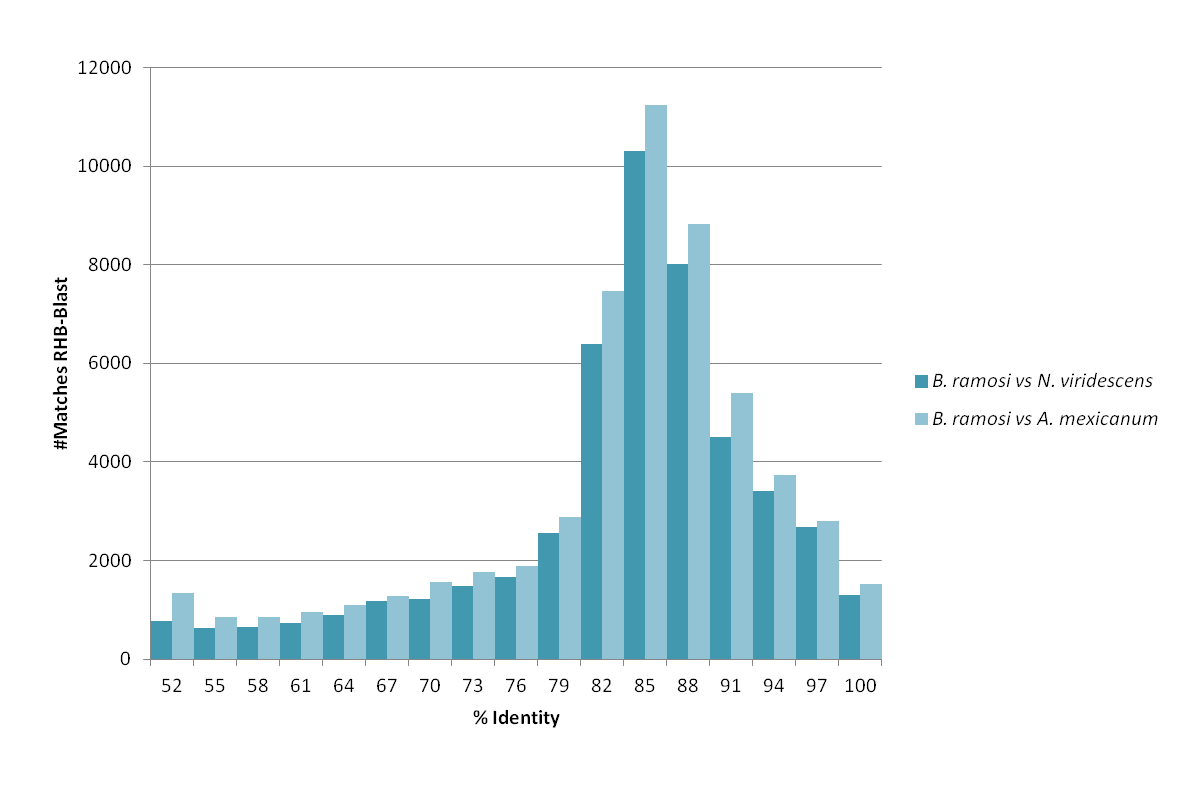

Supplement: Supplementary file 6 — Histogram that shows the frequencies of the % identity between B. ramosi vs N. viridescens and B. ramosi vs A. mexicanum during the RHB-Blast. (TIF 3750 kb) [file 12864_2018_5076_MOESM6_ESM.tif]

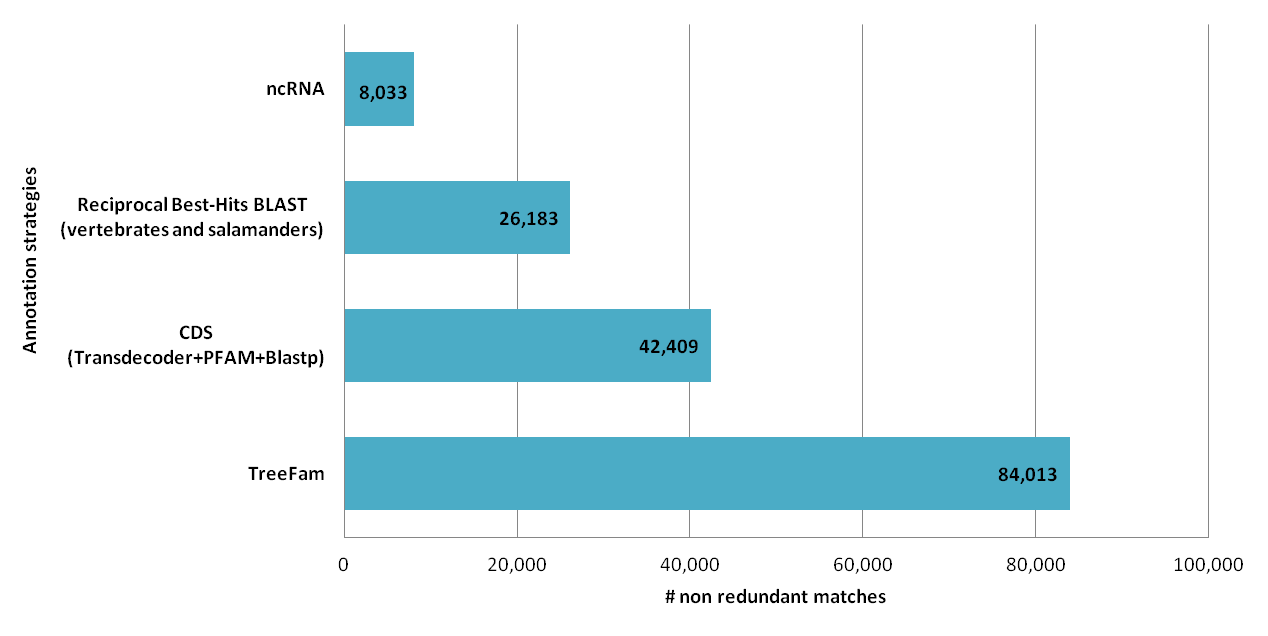

Supplement: Supplementary file 10 — Homology assignments recovered in de novo reference transcriptome assembly of Bolitoglossa ramosi . The B. ramosi transcriptome was surveyed by Reciprocal Best Hits of translated BLAST searches (RBH-BLAST) to protein or translated databases from different vertebrates. Additional gene family homologs were assigned to B. ramosi using protein BLAST against the UniRef90, TreeFam and PFAM domain databases, as well as BLASTN against ncRNA databases. (TIF 48 kb) [file 12864_2018_5076_MOESM10_ESM.tif]
