# Supplementary material for: Using transcriptomics to enable a plethodontid salamander (Bolitoglossa ramosi) for limb regeneration research
Source: BMC Genomics. 2018 Sep 25;19:704. doi: 10.1186/s12864-018-5076-0 (PMC6157048; doi:10.1186/s12864-018-5076-0)
Supplement: Supplementary file 4 — BUSCO Analysis of Transcriptome Completeness. (DOCX 11 kb) [file 12864_2018_5076_MOESM4_ESM.docx]

| *Bolitoglossa ramosi*  transcriptome | BUSCO Data Base Annotation | |
| --- | --- | --- |
|  | Metazoa | C:85.2%[S:79.9%,D:5.3%],F:7.4%,M:7.4%,n:978 |
|  | Eukaryota | C:85.1%[S:79.2%,D:5.9%],F:6.6%,M:8.3%,n:303 |
|  | Vertebrata | C:78.2%[S:75.8%,D:2.4%],F:9.9%,M:11.9%,n:2586 |

Key: C: Complete; D: Duplicated; F: Fragmented; M: Missing; n: Number of BUSCO genes
